# *Streptococcus sanguinis*-induced cytokine and matrix metalloproteinase-1 release from platelets

**DOI:** 10.1186/1471-2172-15-15

**Published:** 2014-04-22

**Authors:** Fabrice Cognasse, Hind Hamzeh-Cognasse, Adrien Chabert, Elke Jackson, Charles-Antoine Arthaud, Olivier Garraud, Archie McNicol

**Affiliations:** 1 Etablissement Français du Sang (EFS) Auvergne-Loire, Saint-Etienne, France; 2 GIMAP-EA3064, Université de Lyon, Saint-Étienne 42023, France; 3 Oral Biology and Pharmacology & Therapeutics, University of Manitoba, Winnipeg, MB, Canada; 4 International Centre for Oral Systemic Health, University of Manitoba, Winnipeg, MB, Canada

**Keywords:** Platelets, Cytokines, Signalling, Oral cavity, Inflammation, *Streptococcus sanguinis*

## Abstract

**Background:**

*Streptococcus sanguinis* (*S.sanguinis*), a predominant bacterium in the human oral cavity, has been widely associated with the development of infective endocarditis. Platelets play both a haemostatic function and can influence both innate and adaptive immune responses. Previous studies have shown that *S.sanguinis* can interact with, and activate, platelets.

**Results:**

The aim of this study was to determine whether *S.sanguinis* stimulates the release of matrix metalloproteinases (MMPs) 1, 2 and 9 and the pro-inflammatory mediators SDF-1, VEGF and sCD40L, from platelets and to subsequently pharmacologically address the release mechanism (s). *S.sanguinis* stimulated the release of MMP-1, SDF-1, VEGF and sCD40L from platelets and inhibitors of cyclooxygenase and phosphatidylinositol 3-kinase, and antagonists of the αIIbβ3 integrin and glycoprotein Ib, each inhibited the secretion of all factors.

**Conclusions:**

Therefore the release of MMP-1, SDF-1, VEGF and sCD40L occurs late in the platelet response to *S.sanguinis* and highlights the complex intracellular signalling pathways stimulated in response to *S.sanguinis* which lead to haemostasis, MMP and pro-inflammatory mediator secretion.

## Background

The oral cavity is one of the most common gateways of entry of bacteria, including *Streptococcus sanguinis* (*S. sanguinis*) an organism which has been implicated in the development of infective endocarditis [1], into the blood [2]. A correlation exists between periodontitis, where there is an increased incidence of bacteremias [3], and the development of cardiovascular disease [4], although the causality is controversial [5]. Multiple studies have demonstrated that *S. sanguinis* can activate human platelets in a strain and donor dependent manner [6], and therefore it is possible that platelets play a central role in any relationship between periodontitis and cardiovascular pathologies [6].

Traditionally platelets have been considered solely as components of haemostatis and by extension the pathological process of thrombosis [7,8]. However, it is now clear that platelets are innate immune cells and are associated with the early stages of atherosclerosis and other inflammatory conditions [9-11]. Platelets contain in, and secrete from, alpha granules a range of soluble immunomodulatory factors such as Stromal cell–derived factor (SDF)-1/CXCL12, a member of the CXC chemokine family and Vascular Endothelial Growth Factor (VEGF), a sub-family of growth factors, stimulate vasculogenesis and angiogenesis [12]. Furthermore, CD40 ligand (CD40L, CD154), of which approximately 95% of its soluble form (sCD40L) is generated from platelets, is a modulator of humoral and cellular immunity, has pro-inflammatory properties and provides a link between the immune system and atherothrombosis [13].

Several recent data, including our own, demonstrate that platelets have the capacity to sense external signals through a single type of pathogen recognition receptor and differentially adjust the innate immune response by the appropriate secretion of a number of cytokines/chemokines and some of their receptors [14,15]. Italiano and colleagues initially demonstrated that there are two discrete sub-populations of platelet alpha granules, one containing pro-angiogenic factors and one containing anti-angiogenic factors [16,17]; indeed subsequent studies have suggested more heterogeneity among these granules [18]. This has led to the concept of differential alpha granule release, although the mechanisms remain unknown [17].

Matrix MetalloProteinases (MMPs) constitute a family of zinc- and calcium-dependent proteinases that are involved in the turnover of the extracellular matrix (ECM) of connective tissue. They degrade most components of the ECM and participate in a variety of pathological processes, including atherosclerosis, myocardial infarction and aortic aneurysms, as well as tumour growth and metastasis [19]. MMP-1 is expressed on the surface of resting platelets and, following platelet activation, its levels are upregulated and its activity engaged [20]. There is less MMP-2 than MMP-1 on the platelet surface, and the presence of both MMP-3 and MMP-9 is controversial [20]. In addition to its effects on the extracellular matrix, MMP-1 can regulate outside-in signalling in platelets resulting in the phosphotyrosine phosphorylation, and subsequent redistribution, of β3 integrins as a pre-requisite for platelet aggregation [20].

Interestingly, collagen can activate MMP-1, which in turn cleaves the platelet protease activating receptor, PAR-1, with the resultant engagement of the receptor and enhanced platelet activation [21]. To date there are no reports of the effects of *S. sanguinis* on MMPs in platelets.

The aim of this study was to determine whether *S. sanguinis* releases platelet MMPs (MMP-1, 2 and 9) and to pharmacologically address the mechanism by which the MMPs and pro-inflammatory mediators (SDF-1, VEGF and sCD40L) are released.

## Results and discussion

**Figure 1:**
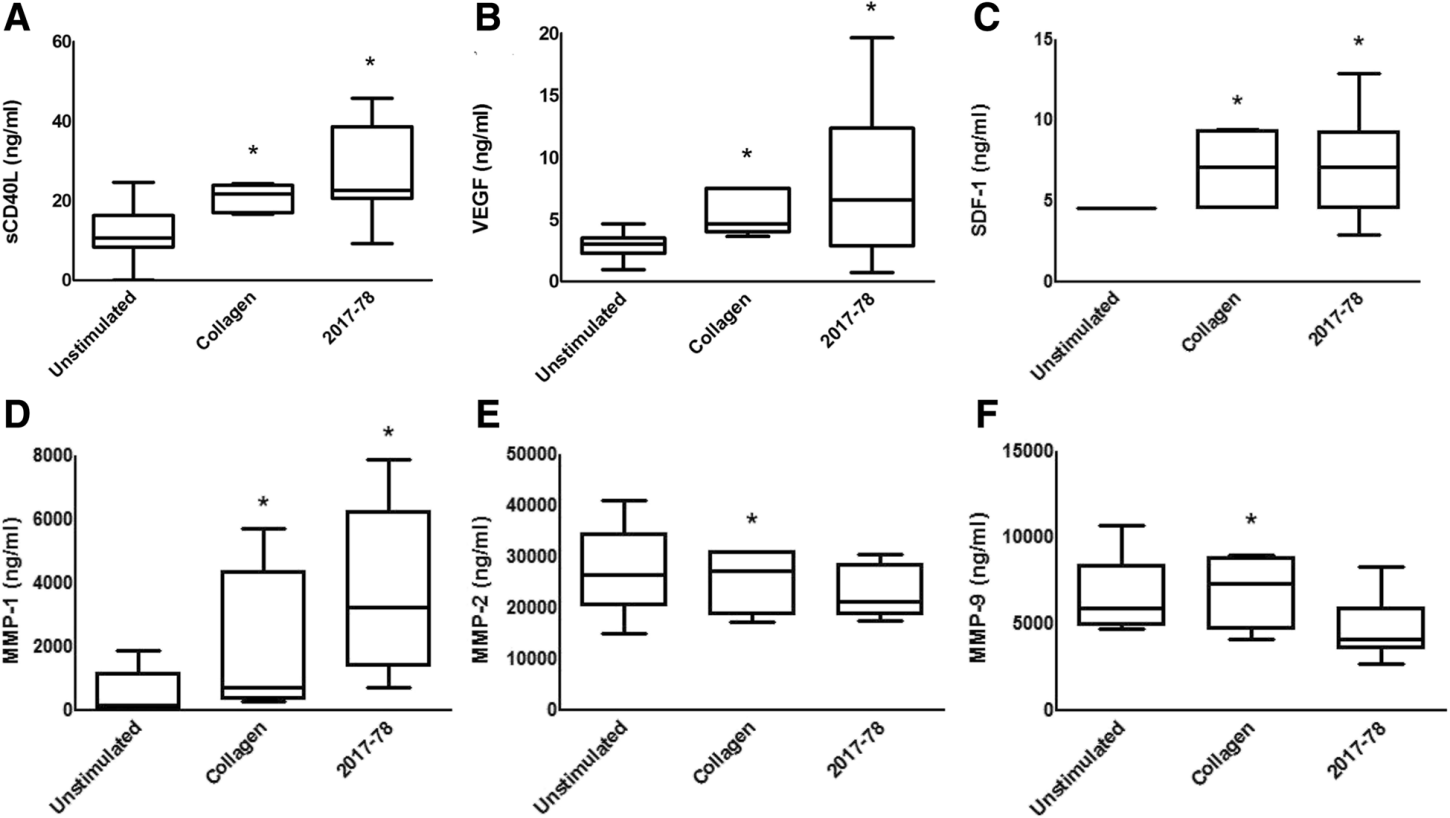
***Streptococcus sanguinis*-induced soluble molecule secretion from platelets.** Platelets were stirred in with either *S. sanguinis* strain 2017–78 (n = 15), collagen (2 μg/ml; n = 5) or saline control (Unstimulated; n = 11). Release reactions were terminated and the levels of **(A)** sCD40L, **(B)** VEGF, **(C)** SDF-1, **(D)** MMP-1, **(E)** MMP-2 and **(F)** MMP-9 in the releasates determined by multi-plex luminex. Results are mean ± SEM of several individual experiments using the platelets from different donors and subsequently analyzed using the Mann–Whitney U-test (^#^P < 0.05 with respect to saline control; *P < 0.05 with respect to the saline control).

Kerrigan and colleagues have suggested that the interaction between *S sanguinis* and GPIb on platelets is not only important for the pathogenesis of infective endocarditis but may also play a contributory role in some cases of myocardial infarction [22]. Studies have shown that plasma levels of sCD40L is a predictor of recurrent cardiovascular disorders (*e.g.* myocardial infarction and stroke) [23]. As previously observed [24], *S. sanguinis* 2017–78 stimulated the release of significant amounts of sCD40L from platelets (Figure 1A), consistent with Kerrigan and colleagues proposed role for platelets in *S sanguinis*-induced myocardial infarction [22]. The secretion of sCD40L was accompanied by the secretion of VEGF (Figure 1B), SDF-1 (Figure 1C) and MMP-1 (Figure 1D). In each case, the level of secretion elicited by *S. sanguinis* 2017–78 was statistically similar to that elicited by the soluble positive control collagen (Figure 1A–D).

In contrast *S. sanguinis* 2017–78 did not elicit the release of MMP-2 (Figure 1E) or MMP-9 (Figure 1F). This constitutes the first demonstration that the oral microorganism *S. sanguinis* can induce the secretion of a tissue-destructive molecule (MMP-1) from platelets to the local vasculature, thus potentially participating in the breakdown of the ECM at sites of vascular lesion. The functional significance of the secretion of MMP-1 is unclear. A novel autocrine role for MMP-1 in collagen-stimulated platelets has been proposed, whereby collagen converts inactive MMP-1 to active MMP-1 which in turn cleaves the thrombin receptor PAR-1 exposing the ligand and leading to full platelet activation [20]. A similar mechanism in *S. sanguinis*-induced platelet activation is attractive as *S. sanguinis* and collagen utilise many similar intracellular signalling pathways [25]. To date the effects of PAR-1 receptor antagonists, such as SCH 530398, on platelet activation induced by either collagen or *S. sanguinis* have not been examined. Interestingly, however, certain strains of *Porphyromonas gingivalis* have been shown to activate platelets via the PAR-1 and PAR-4 thrombin receptors on platelets [26]. Inhibition of this proteolytic action had no effect on this activation, and therefore it is clear that a complex mechanism is involved [27] and a role for MMP-1 cannot be discounted.

The absence of MMP-2 secretion may reflect the subcellular localisation of MMP-2 within the platelet. Studies by Sawicki and colleagues suggest that MMP-2 is localised to the cytosol rather than to granules [28]. Although in the same studies MMP-2 was secreted in activated platelets [28], it is possible that the weak nature of platelet activation by *S. sanguinis* cannot stimulate this release.

The presence, secretion and release of MMP-9 from platelets is controversial. Some studies suggest that platelets do not secrete MMP-9 [29,30], whereas others report, not only that MMP-9 is secreted from platelets *in vivo*, but that it plays a crucial role in sCD40L release in pathological conditions such as Behçet’s disease [31] and abdominal sepsis [32].

To determine the potential roles played by individual signalling pathways in the secretion of sCD40L, VEGF, SDF-1 and MMP-1 in response to *S.sanguinis*, we employed various inhibitors of biochemical pathways at concentrations previously reported to have maximal effects. Such concentrations optimise the conditions to identify individual signalling pathways, although recognising the potential for non-selective actions.

**Figure 2:**
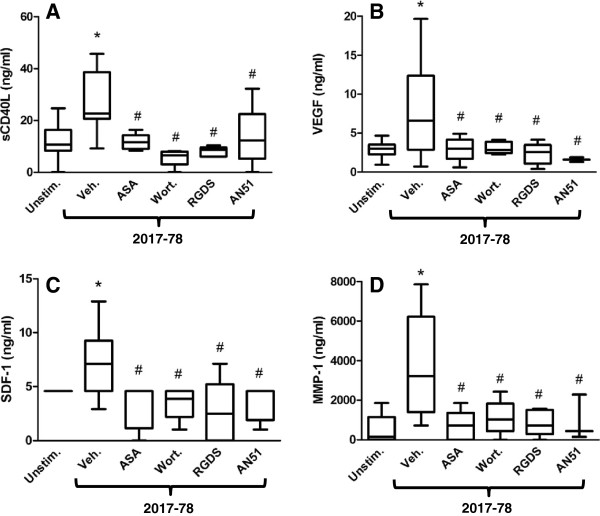
**Effects of inhibitors on *Streptococcus sanguinis *induced sCD40L (A), VEGF (B), SDF-1 (C) and MMP-1 (D) secretion from platelets.** Platelets were pre-incubated with either aspirin (100 μM; 20 mins.; n = 7), wortmannin (100 nM; 2 mins; n = 5), RGDS (1 mM; 5 mins; n = 5), AN51 (0.165 mg/mL, 5 mins; n = 5) or the saline control then stirred with *S. sanguinis* strain 2017–78. Release reactions were terminated and the levels of sCD40L in the releasates determined by multi-plex luminex. Results are shown as box plots with whiskers for the data range representing median, upper and lower quartiles of several individual experiments using the platelets from different donors and subsequently analyzed using the Mann–Whitney U-test (*P < 0.05 with respect to saline control; ^#^P < 0.05 with respect to *S. sanguinis* strain 2017–78).

We have previously shown roles for GPIb, COX, the αIIbβ3 integrin and PI3K in *S. sanguinis*-induced platelet aggregation [25,33,34]. Consistent with the aggregation results [24], pre-treatment of platelets with the GPIb antagonist AN51 (0.165 mg/mL, 5mins.), the COX inhibitor aspirin (100 μM; 20 min), the αIIbβ3 integrin antagonist RGDS (1 mM; 5 mins.) or the PI3K inhibitor wortmannin (100 nM; 2 mins.) each significantly decreased *S. sanguinis* strain 2017-78-induced secretion of sCD40L (Figure 2A).

Pretreatment of platelets with the same inhibitors also decreased the secretion of VEGF (Figure 2B), SDF-1 (Figure 2C) and MMP-1 (Figure 2D) consistent with roles for GPIb, COX, the αIIbβ3 integrin and PI3K in the secretion of these proteins. The precise sublocalisation of SDF-1, VEGF and MMP-1 within the individual heterogenic population of granules is unknown. However, the current observations suggest, either that SDF-1, VEGF and MMP-1 are contained in the same granule population, or that the granule populations containing the proteins are regulated by the same signalling pathways.

## Conclusions

The concept of differing signalling pathways regulating the release of the individual populations of alpha granules was introduced by Italiano and Batinelli [17]. The pathway(s) involved in secretion has not been established. However as secretion was sensitive to inhibition by RGDS, it appears that it occurs distal to the engagement of the αIIbβ3 integrin. Speculatively this could lead to the incorporation, and activation, of ERK into the cytoskeleton [35], Rac activity, cytoskeletal rearrangement [36] and the subsequent alpha granule secretion [37]. Further description of their mechanisms of action will expand our understanding of platelet activation and oral/mucosal inflammatory process and produce new therapeutic strategies.

## Methods

### Materials

*S. sanguinis* strain 2017–78 was a gift from Dr M. Herzberg (University of Minnesota) [38]. Blood Agar Base No. 2 was obtained from Oxoid Inc. (Nepean, ON, Canada) and sheep’s blood from Atlas Laboratories (Winnipeg, MB, Canada). Collagen, the cyclo-oxygenase (COX) inhibitor aspirin, the phosphatidylinositol 3-kinase (PI3K) inhibitor wortmannin, the peptise αIIbβ3 integrin antagonist Argine-Glycine-Aspartic Acid-Serine (RGDS), and the GP (glycoprotein) Ib receptor blocker AN51 were all purchased from Sigma-Aldrich Canada Ltd (Oakville, ON, Canada).

### Blood collection

The study was approved by the Research Ethics Board of the University of Manitoba. Blood was collected after informed consent, from healthy human volunteers who had denied taking medication known to interfere with platelet function. Platelet-rich plasma (PRP) was prepared by centrifugation (200 × g; 20 min). *S. sanguinis* strain 2017–78 was prepared as previously reported [24]. PRP was stirred with *S. sanguinis* strain 2017–78 in the presence of the inhibitor or vehicle control, and was aggregation monitored continuously. At a time corresponding to that which *S. sanguinis* strain 2017–78 induced maximal aggregation, the release was terminated, the sample was centrifuged and the supernatants stored at -80 ºC until assayed [24].

### Soluble factor detection

The levels of soluble factors MMPs (MMP-1, 2 and 9) and pro-inflammatory mediators (SDF-1, VEGF and sCD40L) were measured in duplicate from the releasates by multi-plex luminex kits (Bio-Rad, Marnes-la-Coquette, France). Absorbance at 450 nm was determined using an ELISA reader (Miltiskan EX, Labsystem, Helsinki, Finland), as previously reported [39].

### Statistical analysis

Inter-experiment comparisons of *S. sanguinis* strain 2017-78-induced MMPs and cytokines release were analyzed by means of the paired *t*-test. The effects of inhibitors on *S. sanguinis* strain 2017-78-induced secretion were analyzed using the non-parametric Mann–Whitney *U*-test. Results are shown as box plots with whiskers for the data range representing median, upper and lower quartiles and a *P*-value < 0.05 was considered to be significant.

## Abbreviations

S sanguini: *Streptococcus sanguinis*; MMP: Matrix metalloproteinase; SDF1: Stromal cell–derived factor 1; VEGF: Vascular endothelial growth factor; sCD40L: Soluble CD40 ligand; COX: Cyclooxygenase; PI3K: Phosphatidylinositol 3-kinase; RGDS: Argine-glycine-aspartic acid-serine peptide; GP: Glycoprotein; ECM: Extracellular matrix; PRP: Platelet-rich plasma.

## Competing interests

The authors declare no competing financial interests and no conflict of interest regarding this study.

## Authors’ contributions

FC, HHC, AMcN, OG: Analysis and interpretation of results; FC, CAA, HHC, AC, EJ, AMcN, OG; Preparation of manuscript. All authors read and approved the final manuscript.
